# Nutritional Value and Bioactive Compounds Characterization of Plant Parts From *Cynara cardunculus* L. (Asteraceae) Cultivated in Central Greece

**DOI:** 10.3389/fpls.2018.00459

**Published:** 2018-04-10

**Authors:** Spyridon A. Petropoulos, Carla Pereira, Nikolaos Tzortzakis, Lillian Barros, Isabel C. F. R. Ferreira

**Affiliations:** ^1^Laboratory of Vegetable Production, University of Thessaly, Magnissia, Greece; ^2^Centro de Investigação de Montanha, Instituto Politécnico de Bragança, Bragança, Portugal; ^3^Department of Agricultural Sciences, Biotechnology and Food Science, Cyprus University of Technology, Lemesos, Cyprus

**Keywords:** antioxidant activity, caffeoylquinic acids, cardoon, flavonoids, nutritional value, phenolic compounds, proximate analysis, seed oils

## Abstract

In the present study, the nutritional value of the edible parts (immature capitula) of cardoon plants was evaluated, while further analyses were carried out in order to assess antioxidant properties and phenolic compounds composition of the various plant parts and seed oils. Cardoon capitula (heads) were a rich source of carbohydrates, with the main detected free sugar being sucrose, as well as of macro- and micro-minerals (K, Ca, Mg, and Fe). Heads were also abundant in saturated fatty acids (palmitic, behenic, linoleic, stearic, caproic, and oleic acid), whereas seed oils in unsaturated fatty acids (linoleic, oleic, palmitic, and stearic acid). Total phenolic compounds (TPC) content and phenolics composition differed between the various plant parts, with heads and leaf blades having higher TPC than midribs and petioles. Moreover, heads and leaf midribs and petioles consisted mainly of phenolic acids (5*-O-*caffeoylquinic and 3,5-*O*-dicaffeoylquinic acid), with flavonoids being detected in lower amounts. In contrast, the composition of polyphenols in leaf blades consisted mostly of flavonoids (Luteolin-7-*O*-glucoside and luteolin-7-*O*-malonylhexoside), whereas phenolic acids were also detected in considerable amounts (5-*O*-feruloylquinic and 3-*O*-caffeoylquinic acid). Regarding antioxidant properties, leaf blades and seeds exhibited the highest potency for all the tested assays which could be partly attributed to the synergistic effects of the phenolic compounds present in each sample. In conclusion, cardoon plant parts may find various uses in the food and pharmaceutical industry, since they contain considerable amounts of bioactive molecules, while seed oils can be considered as alternative vegetable oils for human consumption.

## Introduction

*Cynara cardunculus* L. (Asteraceae) is a species native in the Mediterranean basin, which shows great adaptation ability in various soil and climate conditions and abiotic stress factors, including high salinity levels and water deficit (Benlloch-González et al., 2005; Ceccarelli et al., 2010; Pagnotta et al., 2017). The species includes globe artichoke [*C. cardunculus* var. *scolymus* (L.) Fiori] and cardoon or leafy cardoon, which is further divided in cultivated cardoon (*C. cardunculus* var. *altilis* DC) and its wild ancestor [wild cardoon: *C. cardunculus* var. *sylvestris* (Lamk) Fiori; (Rottenberg and Zohary, 1996; Raccuia et al., 2004; Pagnotta et al., 2017)]. Cultivated cardoon is a perennial field crop which during the last years has been suggested as an alternative energy crop due to its low crop requirements and high annual biomass production, as well its high heating value (Foti et al., 1999). The annual biomass production (excluding seeds) ranges between 10 and 20 t ha^−1^, depending on soil and climate conditions, while energy value can be as high as 15 MJ kg^−1^ (Raccuia and Melilli, 2007; Angelini et al., 2009).

The edible part of both wild and cultivated species is the immature capitula (flowering heads), which are used in many local dishes throughout the Mediterranean basin, as well as the tender inner stalks and leaf petioles which are usually consumed as cooked or salad vegetables (Fernández et al., 2006; Christaki et al., 2012). Moreover, the whole plant may be used for medicinal and industrial purposes, as well as in the food industry as natural rennet for cheese production (Fernández et al., 2006; Borgognone et al., 2014).

Apart from energy production and food purposes, cardoon plant parts can be also a high added value product since they are a rich source of bioactive compounds which can be used in the pharmaceutical and nutraceutical industry, while seed oils can be used not only for biofuel production but also for human consumption due to its high nutritional value (Curt et al., 2002; Fernández et al., 2006; Raccuia and Melilli, 2007). According to Raccuia et al. (2011) who evaluated various domesticated and wild types of cardoon, cardoon seed oil is a rich source of unsaturated fatty acids such as linoleic and oleic acids (44.5 and 42.6%, respectively), whereas saturated fatty acids such as palmitic and stearic acid were detected in lower amounts (9.8 and 3.1%, respectively). In addition, the same authors observed a great variation in fatty acids composition between the tested genotypes with unsaturated acids being more abundant than saturated for all the genotypes (Raccuia et al., 2011).

Several studies have confirmed the high bioactive compounds content and antioxidant potency of the species (Valentāo et al., 2002; Kukić et al., 2008; Durazzo et al., 2013; Kollia et al., 2016). According to Borgognone et al. (2014), cardoon leaves contain higher amounts of total phenolic compounds and flavonoids and exhibit a higher antioxidant potency than artichoke leaves, while the main detected polyphenols in cardoon leaves were chlorogenic acid, cynarin and caffeoylquinic acid derivatives, luteolin, and derivatives (Pandino et al., 2010). Moreover, according to the same study growth stage and salinity stress have a pivotal role on bioactive compounds composition, with highest contents of chlorogenic acid and cynarin being observed at 105 days after sowing (DAS) while luteolin and its derivative on 82 DAS (Borgognone et al., 2014). Other factors that may affect chemical composition and recovery of bioactive compounds from cardoon leaves include nitrogen availability (Borgognone et al., 2016), extracting process (Brás et al., 2015), plant part (Falleh et al., 2008; Pandino et al., 2011b), nutrient solution composition (Rouphael et al., 2012) and salinity stress (Colla et al., 2013) among others.

Cardoon is an important field crop of the Mediterranean basin with high potential for industrial uses such as energy and solid biofuel production. However, although the wild ancestor of the species has been traditionally used for human consumption with similar uses as globe artichoke, there is scarce literature regarding the nutritional value of the species. Therefore, the aim of the present study was to evaluate the nutritional value of cultivated cardoon heads cultivated in central Greece, as well as the potential of using various plant parts as sources of bioactive compounds. Considering the high content of the species in bioactive molecules with significant medicinal and therapeutic properties, we performed the characterization of the various plant parts, including leaves (blades and petioles), heads, stems and seeds in terms of chemical composition, with a special focus on phenolic compounds content. Finally, we evaluated the antioxidant properties of plant parts with various assays in order to assess the antioxidant potency of the species.

## Materials and methods

### Plant material and sampling

**Figure 1:**
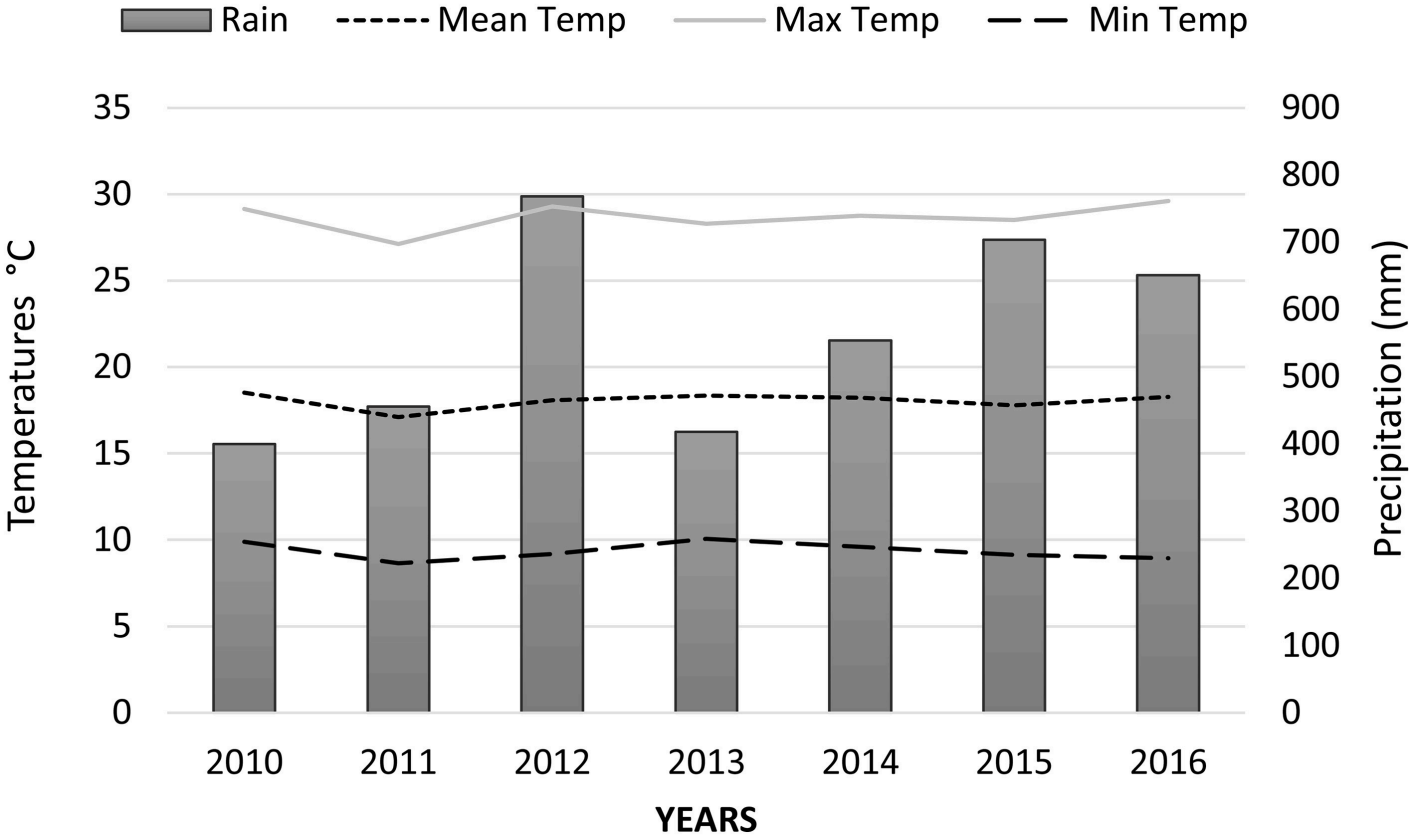
Yearly precipitation (mm) and temperatures (°C) at the experimental site from the start of crop establishment (2010–2016).

**Figure 2:**
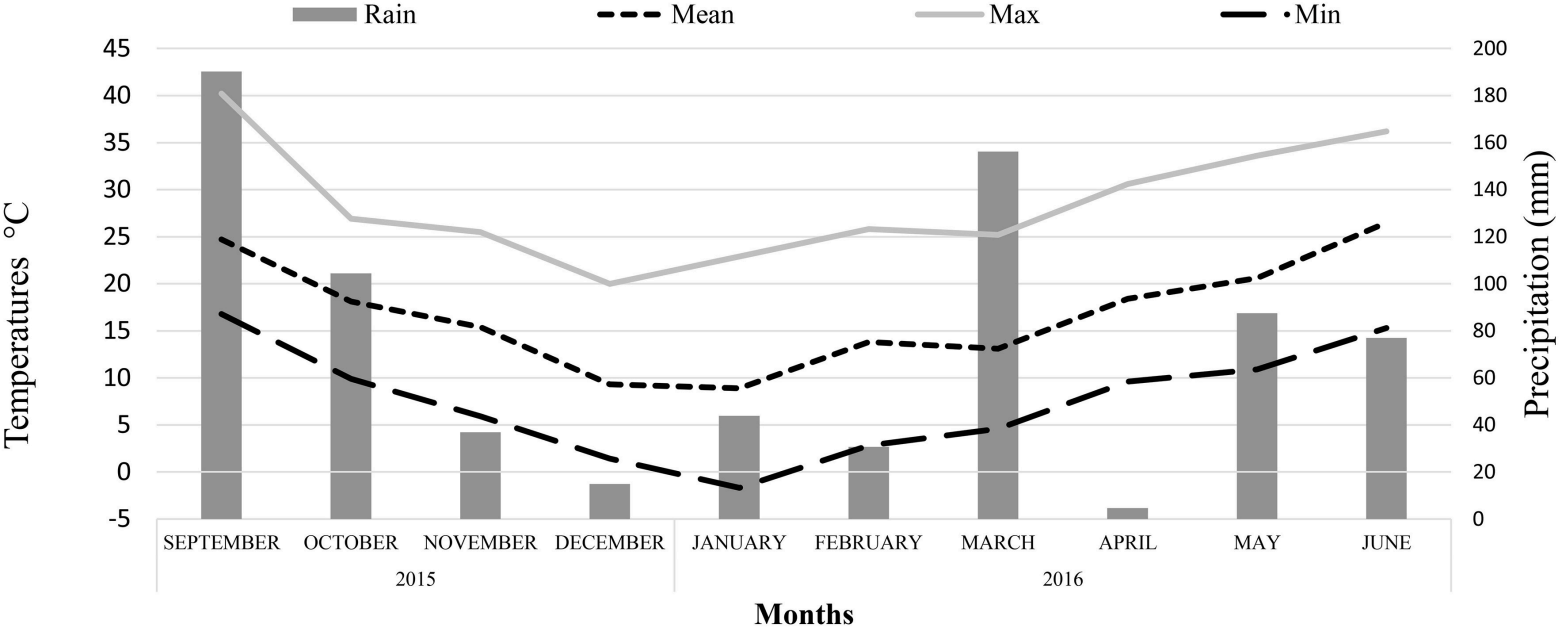
Monthly precipitation and temperatures (°C) at the experimental site during the harvesting period (September 2015–June 2016).

Field experiments were carried out at the experimental farm of the University of Thessaly in Velestino, Greece during the growing period of 2015–2016 (January–June). Samples of cultivated cardoon [*Cynara cardunculus* L. var. *altilis* DC] cv. Biango Avorio (Fratelli Ingegnoli Spa, Milano, Italy) were collected from plants grown from seeds, 5 years after crop establishment. Soil analyses were performed prior to crop establishment and soil composition was the following: 48% Sand; 29% Silt; 23% Clay; 1.3%; Organic matter; pH 7.9; EC: 1.4 mS cm^−1^; ${\text{NO}}_{3}^{-}$: 9.49 mg kg^−1^; P: 74.53 mg kg^−1^; K_exch_: 0.98 cmol_c_ kg^−1^; Ca_exch_: 13,96 cmol_c_ kg^−1^; Mg: 4,32 cmol_c_ kg^−1^. Fertilization was applied with basal dressing prior to seeding, supplying 50 kg ha^−1^ N, 90 kg ha^−1^ P_2_O_5_ and 40 kg ha^−1^ K_2_O_._ After crop establishment, nitrogen fertilizers were applied with side dressing at each growing period and before plant regrowth (100 kg ha^−1^ N). Plant density was 40,000 ha^−1^ with distances of approximately 0.6 m between rows and 0.4 m within rows. Irrigation was applied monthly during the first growing period (staring on April and until July) with water cannons, whereas in the following years irrigation was applied only twice in each growing period (on April and May) due to the extensive root system that plants form after the second year of establishment. Weed control was applied with hoeing after plant regrowth at each growing period, since at later growth stages plant is very competitive against weeds. No pesticides and fungicides were applied. Climate data from crop establishment and during the harvesting period are presented in Figures 1, 2, respectively.

Samples of leaves were collected on April 10th from 15 individual plants (one leaf per plant; *n* = 15) at principal growth stage 4 (stage code 49; prior to stem elongation from the latest fully developed leaves) according to phenological stages description of cardoon by Archontoulis et al. (2013), and further separated into blades, and petioles and midribs. Batch samples of leaves were prepared as previously described by the authors (Petropoulos et al., 2017b). Briefly, after leaves separation in midribs, petioles and blades, all samples were chopped, lyophilized and put in air sealed bags and at deep freezing conditions (−80°C) until further analyses. Heads were collected on May 5th from 15 individual plants (one head per plant; *n* = 15) at principal growth stage 5 (code stage 53) and when all heads reached full size and before being inedible and obtain hard texture (Archontoulis et al., 2013). Seeds were collected on June 26th from 15 individual mature heads (one mature head per plant; *n* = 15) at principal growth stage 8 (code stage 89) and as soon as they were open, brown-yellow, dry and senesced (Archontoulis et al., 2013). Batch samples from heads and seeds were prepared as previously described by the authors (Petropoulos et al., 2017a, in Press). Briefly, heads were chopped and inedible parts were removed in order to keep only the inner part of the head. For seed collection, mature heads were cut and cleared from bracts, sepals and petals in order to make seeds visible and able to remove them from the receptacle.

### Nutritional value analysis

Head samples were analyzed in terms of macronutrients (moisture, proteins, fat, carbohydrates, and ash), according to the AOAC procedures (AOAC, 2016). Crude protein content (N × 6.25) was estimated using the macro-Kjeldahl method; Soxhlet extraction with petroleum ether was used to determine the crude fat content; incineration at 600 ± 15°C was used to measure ash content. Total carbohydrates were calculated by difference and the energetic value was calculated as follows: Energy (kcal) = 4 × (g protein + g carbohydrate) + 9 × (g fat).

Free sugars of heads analysis was performed by high-performance liquid chromatography with a refraction index detector (HPLC-RI; Knauer, Smartline system 1000, Berlin, Germany), as previously described by Guimarães et al. (2013). The sugars were identified by comparing their retention times with standard compounds and quantification was conducted by using the internal standard (IS, melezitose) methodology.

### Chemical composition analysis

Fatty acids of heads were analyzed with a DANI 1,000 gas chromatographer (GC) coupled to a flame ionization detector (FID) after a transesterification procedure described by Guimarães et al. (2013) and results were recorded and processed using Clarity 4.0.1.7 Software (DataApex, Podohradska, Czech Republic).

Seed oils were obtained by a screw type small size press (Täby Pressen, Type 40; Örebro, Sweden), while sampling was carried out in triplicate from batch samples of 1,000 g seeds. Press-extracted oils were centrifuged twice (3,500 × g for 10 min at 25°C) and the supernatant was put in an amber screw cap vial and stored in a desiccator under dark conditions and room temperature (24 ± 1°C) until analysis. Fatty acids composition was performed with the above-described methods for heads (Guimarães et al., 2013).

For mineral composition analyses, fresh plant tissues samples (heads, leaf blades, midribs and petioles, and seeds) were dried in a forced-air oven at 72°C to constant weight, then ground to a powder with a domestic coffee seeds grinder. The ground samples were subjected to dry ashing at 500°C and extracted with 1 N HCl to determine the mineral composition. Ca, Fe, Mg, Mn, and Zn content were determined by atomic absorption spectrophotometry (Perkin Elmer 1,100B, Waltham, MA), while Na and K content by flame photometry (Sherwood Model 410, Cambridge, UK).

### Phenolic compounds characterization

For phenolic compounds composition of plant parts (heads, leaf blades, midribs and petioles, and seeds), extracts were prepared by stirring the dry sample (1 g) and 30 mL of methanol/water (80:20 v/v, at 25°C at 150 rpm) for 1 h and afterwards filtered using Whatman paper No. 4. The residue was then extracted with an additional portion of (30 mL) methanol/water and the combined extracts were evaporated under reduced pressure (rotary evaporator Büchi R-210, Flawil, Switzerland), until complete removal of methanol. The aqueous phase was frozen and lyophilized (FeeeZone 4.5, Labconco, Kansas City, MO, USA).

The hydroalcoholic extracts were re-dissolved in methanol/water (80:20 v/v) to a final concentration of 2 mg/mL for phenolic compound identification and quantification. LC-DAD–ESI/MSn analyses were performed for phenolic compounds identification and quantification, using a Dionex Ultimate 3000 UHPLC instrument (Thermo Scientific, San Jose, CA, USA) equipped with a diode-array detector and coupled to a mass detector, following a procedure previously reported by Bessada et al. (2016). The chromatogram was recorded at several wavelengths, characteristic of different classes of polyphenols, such as 280 nm for phenolic acids (non hydroxycinnamic acids derivatives) and flavonones, 330 nm for hydroxycinnamic acids derivatives and 370 nm for flavones. For quantitative analysis, a calibration curve (200-5 μg/mL) for each available phenolic standard was constructed based on the UV signal. For the identified phenolic compounds for which a commercial standard was not available, the quantification was performed through the calibration curve of the most similar available standard. The results were expressed as mg/g of extract.

### Antioxidant activity evaluation

The same extracts from the phenolic characterization were re-dissolved in methanol/water (80:20, v/v) in order to be submitted to distinct *in vitro* antioxidant activity assays, at a final concentration of 20 mg mL^−1^ and further diluted to different concentrations. For the purposes of the study four *in vitro* assays were performed as previously described by Petropoulos et al. (2015).

In particular, the 2,2-diphenyl-1-picrylhydrazyl (DPPH) radical-scavenging activity was evaluated by using an ELX800 microplate reader (Bio-Tek Instruments, Inc.; Winooski, VT, USA), and calculated as a percentage of DPPH discoloration using the formula: [(A_DPPH_-A_S_)/A_DPPH_] × 100, where A_S_ is the absorbance of the solution containing the sample at 515 nm, and A_DPPH_ is the absorbance of the DPPH solution. Reducing power was evaluated by the capacity to convert Fe^3+^ to Fe^2+^, measuring the absorbance at 690 nm in the microplate reader mentioned above. Inhibition of β-carotene bleaching was evaluated through the β-carotene/linoleate assay; the neutralization of linoleate free radicals avoids β-carotene bleaching, which is measured by the formula: β-carotene absorbance after 2 h of assay/initial absorbance) × 100. Lipid peroxidation inhibition in porcine brain homogenates was evaluated by the decreasing in thiobarbituric acid reactive substances (TBARS); the color intensity of the malondialdehyde-thiobarbituric acid (MDA-TBA) was measured by its absorbance at 532 nm; the inhibition ratio (%) was calculated using the following formula: [(A – B)/A] × 100%, where A and B were the absorbance of the control and the sample solution, respectively. The results were expressed in EC_50_ values (sample concentration providing 50% of antioxidant activity or 0.5 of absorbance in the reducing power assay) for antioxidant activity and Trolox was used as a positive control.

### Statistical analysis

For all the applied analytical methodologies, three samples were analyzed for each treatment, whereas all the assays were carried out in triplicate (*n* = 9). The results were expressed as mean values and standard deviations (SD), and analyzed using one-way analysis of variance (ANOVA) followed by Tukey's HSD test with *p* = 0.05. When only two samples were present a Student's *t*-test was used to determine the significant difference between two different samples, with α = 0.05. This analysis was carried out using SPSS v. 23.0 program (IBM Corp., Armonk, NY, USA).

Principal Component Analysis (PCA) was performed in order to examine the contribution of each variables to the total diversity and classify the studied plant parts according to their chemical composition and nutritional value by using statistical program Statgraphics 5.1.plus (Statpoint Technologies, Inc., VA, USA).

## Results and discussion

### Nutritional value

Nutritional value of cardoon heads is presented in Table 1. The edible part of the species has a high water content (81.2%) and low content of fat (0.59 g 100 g^−1^ fw), while it is a rich source of carbohydrates (13.76 g 100 g^−1^ fw) and calorific values (72.4 kcal 100 g^−1^). Moreover, the main detected sugars were sucrose (0.42 g 100 g^−1^ fw), followed by glucose and fructose which were detected in similar amounts (0.09 and 0.10 g 100 g^−1^ fw, respectively).

**Table 1:** Nutritional value and free sugars content of cardoon heads (g 100 g^−1^ fw and kcal 100 g^−1^ for energy; mean values ± SD, *n* = 3).

**Nutritional value**	**Moisture**	**Fat**	**Ash**	**Protein**	**Carbohydrates**	**Energy**
	81 ± 2	0.59 ± 0.01	1.54 ± 0.06	3.03 ± 0.02	13.76 ± 0.04	72.4 ± 0.1
Free sugars	Fructose	Glucose		Sucrose	Total	
	0.09 ± 0.01	0.10 ± 0.02		0.42 ± 0.02	0.61 ± 0.02	

### Chemical composition

Fatty acids composition of cardoon heads and seed oils is presented in Table 2, while seed oil content was 24.5 ± 1.1% (w/w) on a dry weight basis (data not shown). Twenty-two different fatty acids were detected in cardoon heads, while seed oils had a slightly less variable composition with 18 different fatty acids being detected. The main detected fatty acids differed between the studied plant parts, with palmitic and behenic acid being the most abundant fatty acids in heads (47.2 and 10.7%, respectively), followed by linoleic (7.8%), stearic (6.7%), caproic (6.4%), myristic (5.3%), and oleic acid (4.6%). For seed oils, fatty acids composition differed significantly, with linoleic and oleic acids being detected in the highest amounts (64.86 and 21.11%, respectively, while palmitic and stearic acids were found in lower amounts (9.37 and 2.78%, respectively).

**Table 2:** Fatty acids composition of cardoon heads and seed oils (%; mean values ± SD, *n* = 3).

**%**	**Heads**	**Seed oils**	**Student's *t*-test *p*-value**
C6:0	6.4 ± 0.4	0.009 ± 0.001	<0.001
C8:0	1.60 ± 0.07	nd	–
C10:0	1.66 ± 0.04	nd	–
C11:0	0.14 ± 0.01	nd	–
C12:0	1.72 ± 0.01	nd	–
C14:0	5.32 ± 0.03	0.094 ± 0.003	<0.001
C14:1	0.41 ± 0.03	nd	–
C15:0	0.98 ± 0.01	0.021 ± 0.001	<0.001
C16:0	47.2 ± 0.6	9.37 ± 0.09	<0.001
C16:1	0.55 ± 0.02	0.107 ± 0.003	<0.001
C17:0	0.51 ± 0.02	0.072 ± 0.003	<0.001
C18:0	6.7 ± 0.1	2.78 ± 0.01	<0.001
C18:1n9	4.64 ± 0.02	21.11 ± 0.02	<0.001
C18:2n6c	7.76 ± 0.07	64.86 ± 0.07	<0.001
C18:3n3	0.52 ± 0.04	0.108 ± 0.005	<0.001
C20:0	1.27 ± 0.01	0.277 ± 0.003	<0.001
C20:1	nd	0.11 ± 0.03	–
C20:3n3+C21:0	nd	0.380 ± 0.001	–
C20:5n3	0.20 ± 0.01	0.082 ± 0.007	<0.001
C22:0	10.7 ± 0.5	0.461 ± 0.001	<0.001
C22:1n9	0.08 ± 0.01	0.008 ± 0.001	<0.001
C22:2CIS13	0.04 ± 0.01	nd	–
C22:6n3	0.15 ± 0.01	nd	–
C23:0	nd	0.022 ± 0.007	–
C24:0	1.45 ± 0.05	0.121 ± 0.007	<0.001
SFA	85.7 ± 0.2	13.23 ± 0.08	<0.001
MUFA	5.68 ± 0.03	21.34 ± 0.02	<0.001
PUFA	8.7 ± 0.1	65.43 ± 0.07	<0.001
PUFA/SFA	0.10 ± 0.001	4.94 ± 0.07	<0.001
n-6/n-3	9.0 ± 0.4	0.57 ± 0.07	<0.001

*C6:0, caproic acid; C8:0, caprylic acid; C10:0, capric acid; C11:0, undecanoic acid; C12:0, lauric acid; C14:0, myristic acid; C14:1, myristoleic acid; C15:0, pentadecanoic acid; C16:0, palmitic acid; C16:1, palmitoleic acid; C17:0, heptadecanoic acid; C18:0, stearic acid; C18:1n9, oleic acid; C18:2n6c, linoleic acid; C18:3n3, α-linolenic acid; C20:0, arachidic acid; C20:1, eicosenoic acid; C20:3n3+C21:0 cis-11,14,17, eicosatrienoic acid and heneicosanoic acid; C20:5n3, eicosapentaenoic acid; C22:0, behenic acid; C22:1n9, erucic acid; C22:2CIS13, docosadienoic acid; C22:6n3, docosahexaenoic acid; C23:0, tricosanoic acid; C24:0, lignoceric acid; SFA, saturated fatty acids; MUFA, monounsaturated fatty acids; PUFA, polyunsaturated fatty acids; n6/n3, ratio of omega 6/omega 3 fatty acids; nd, not detected*.

Regarding the fatty acid profile, heads and seed oils differed significantly with saturated (SFA) and polyunsaturated (PUFA) fatty acids being the most abundant type of fats in heads and seed oils, respectively (Table 2). Moreover, the fatty acids profile differences had a significant impact on the ratio of n-6/n-3 (9.0 and 0.57 for heads and seed oils, respectively) and PUFA/SFA (0.10 and 4.94 for heads and seed oils, respectively), both of which are associated with the nutritional value and functional properties of a food product.

Mineral composition of the various plant parts is presented in Table 3. The results show that all plant parts exhibited high nutritional value with differences between plant parts regarding their content of the determined minerals. In particular, leaves and heads are rich sources of K (2,800, 3,000, and 2,400 mg 100 g^−1^ dw for heads, leaf midribs and petioles, and leaf blades, respectively) and Ca (1,199, 2,119, and 2,662 mg 100 g^−1^ dw for heads, leaf midribs and petioles, and leaf blades, respectively), while leaf midribs and petioles are rich in Na (1,853 mg 100 g^−1^ dw) and leaf blades in Fe (23 mg 100 g^−1^ dw). Moreover, seeds had a lower content of K (653 mg 100 g^−1^ dw) and Na (18 mg 100 g^−1^ dw) than the other plant parts and similar Mn content with leaf blades (6.0 and 6.3 mg 100 g^−1^ dw, respectively), while a high content of Mg (483 mg 100 g^−1^ dw) was detected in seeds.

**Table 3:** Mineral composition of cardoon heads, leaf midribs and petioles, leaf blades, and seeds (mg 100 g^−1^ dw; mean values ± SD, *n* = 3).

**Plant part**	**K**	**Na**	**Ca**	**Mg**	**Mn**	**Fe**	**Zn**
Heads	2800 ± 145b	240 ± 34c	1199 ± 156c	389 ± 15b	5.5 ± 0.3b	12.2 ± 0.2c	1.17 ± 0.06b
Leaf midribs and petioles	3000 ± 346a	1853 ± 266a	2119 ± 90b	285 ± 45c	3.5 ± 0.3c	13.0 ± 0.6bc	0.15 ± 0.04d
Leaf blades	2400 ± 200c	1027 ± 479b	2662 ± 125a	191 ± 16d	6.3 ± 0.2a	23 ± 2a	0.54 ± 0.06c
Seeds	653 ± 58d	18 ± 4.0bd	1197 ± 195c	483 ± 62a	6.0 ± 0.2a	13 ± 3bc	3.95 ± 0.16a

*In each column, different letters mean significant differences between samples (p < 0.05)*.

### Phenolic compounds

Phenolic compounds characteristics, tentative identification, and quantification for the various plant parts (heads, leaf blades, leaf midribs and petioles, and seeds) are presented in Table 4. The studied plant parts showed significant differences in phenolic compounds content and composition, with heads and leaf blades having the highest total phenolic content (TPC) (80.0 and 63.2 mg g^−1^ extract, respectively). However, heads phenolic compounds consisted mostly of phenolic acids (93.8% of TPC), while in leaf blades flavonoids and phenolic acids were detected in significant amounts (34.4 and 28.7 mg g^−1^ extract, respectively). The main compounds in cardoon heads were *trans* 3,5-*O*-dicaffeoylquinic acid and 5-*O*-caffeoylquinic, while the remaining phenolic compounds consisted mostly of caffeoylquinic acid and apigenin derivatives in a total of seven identified compounds.

**Table 4:** Retention time (Rt), wavelengths of maximum absorption in the visible region (λ_max_), mass spectral data and tentative identification, and phenolic compounds quantification in cardoon heads, leaf blades, leaf midribs and petioles, and seeds (mg g^−1^ extract; mean values ± SD, *n* = 3).

**Peak**	**Rt (min)**	**λ_max_ (nm)**	**Molecular ion [M-H]^−^ (*m/z*)**	**Main MS^2^ fragments (*m/z*)**	**Tentative identification**	**Heads**	**Leaf blades**	**Leaf midribs and petioles**	**Seeds**
1	4.6	328	353	191 (100), 179 (40), 173 (3), 161 (10), 135 (8)	*cis* 3*-O-*Caffeoylquinic acid^1^	nd	nd	0.663 ± 0.001	nd
2	4.9	328	353	191 (100), 179 (46), 173 (3), 161 (8), 135 (6)	*trans* 3*-O-*Caffeoylquinic acid^1^	4.38 ± 0.02a	2.00 ± 0.09b	0.55 ± 0.01c	nd
3	6.1	326	341	179 (100)	Caffeic acid hexoside^2^	nd	0.53 ± 0.06	nd	nd
4	6.8	310	325	163 (100)	*p*-Coumaric acid hexoside^3^	nd	0.60 ± 0.01	nd	nd
5	7.4	326	353	191 (100), 179 (34), 173 (16), 161 (18), 135 (23)	5*-O-*Caffeoylquinic acid^1^	27.8 ± 0.3a	13.38 ± 0.04b	10.6 ± 0.1c	4.25 ± 0.02d
6	11.3	328	515	353 (82), 335 (41), 191 (41), 179 (56), 173 (95), 161 (16), 135 (26)	1,3*-O-*Dicaffeoylquinic acid (cynarin)^1^	nd	nd	0.35 ± 0.01	nd
7	13.3	342	503	461 (100), 447 (23), 285 (23)	Luteolin-*O*-acetylglucuronide^4^	0.885 ± 0.001	nd	nd	nd
8	14.0	325	367	193 (12), 191 (100), 173 (6), 143 (3), 134 (5)	5*-O-*Feruloylquinic acid^5^	nd	0.43 ± 0.03	nd	nd
9	16.5	280	463	287 (100)	Eriodictyol-*O*-glucuronide^6^	nd	nd	0.442 ± 0.001	nd
10	18.4	343	593	285 (100)	Luteolin-7-*O*-rutinoside^4^	nd	tr	0.889 ± 0.007	nd
11	18.8	324	515	353 (82), 335 (41), 191 (41), 179 (56), 173 (95), 161 (16), 135 (26)	*cis* 3,4*-O-*Dicaffeoylquinic acid^1^	1.67 ± 0.09	nd	0.610 ± 0.002	nd
12	18.9	346	461	285 (100)	Luteolin-7-*O*-glucuronide^4^	nd	14.7 ± 0.3	nd	nd
13	19.2	328	515	353 (76), 335 (39), 191 (52), 179 (80), 173 (98), 161 (14), 135 (32)	*trans* 3,4*-O-*Dicaffeoylquinic acid^1^	0.92 ± 0.01	nd	nd	nd
14	19.4	344	447	285 (100)	Luteolin-7-*O*-glucoside^4^	nd	10.5 ± 0.4*	1.35 ± 0.01*	nd
15	20.1	227,279	519	357 (100), 343 (9), 151 (50), 136 (4)	Pinoresinol-4-*O*-hexoside^4^	nd	3.0 ± 0.1	nd	nd
16	20.4	327	515	353 (94), 335 (3), 191 (100), 179 (90), 173 (10), 161 (6), 135 (40)	*cis* 3,5-*O*-Dicaffeoylquinic acid^1^	nd	nd	0.54 ± 0.03	nd
17	21.0	327	515	353 (94), 335 (3), 191 (100), 179 (90), 173 (10), 161 (6), 135 (40)	*trans* 3,5-*O*-Dicaffeoylquinic acid^1^	40 ± 2b	11.80 ± 0.06c	5.0 ± 0.3d	44.71 ± 0.05a
18	22.5	327	515	353 (94), 335 (3), 191 (100), 179 (90), 173 (10), 161 (6), 135 (40)	4,5-*O*-Dicaffeoylquinic acid^1^	nd	nd	0.40 ± 0.01	nd
19	23.3	329	615	515 (12), 453 (100), 353 (56), 335 (3), 191 (3), 179 (3)	Succinoyl-di-*O*-caffeoylquinic acid^1^	nd	nd	1.60 ± 0.05	nd
20	23.3	336	445	269 (100)	Apigenin-*O*-glucuronide^4^	4.8 ± 0.2	nd	nd	nd
21	24.3	347	533	489 (100), 285 (50)	Luteolin-7-*O*-malonylglucoside^4^	nd	6.2 ± 0.3*	1.39 ± 0.03*	nd
TPA						75 ± 2a	28.7 ± 0.2c	20.33 ± 0.06d	48.96 ± 0.03b
TF						5.7 ± 0.2b	31.5 ± 0.3a	4.07 ± 0.02b	nd
OPC						–	3.0 ± 0.1	–	–
TPC						80 ± 2a	63.2 ± 0.4b	24.40 ± 0.07d	48.96 ± 0.03c

Phenolic compound used for quantification: ^1^5-O-caffeoylquinic acid (y = 168823x + 161172; R^2^ = 0.999); ^2^caffeic acid (y = 388345x+406369; R^2^ = 0.994); ^3^p-coumaric acid (y = 301950x+6966.7; R^2^ = 0.999); ^4^apigenin-7-O-glucoside (y = 10683x−45794; R^2^ = 0.996); ^5^ferulic acid (y = 633126x−185462; R^2^ = 0.999); ^6^taxifolin (y = 203766x−208383; R^2^ = 0.999). tr, traces; nd, not detected; TPA, total phenolic acids; TF, total flavonoids; OPC, other phenolic compounds; TPC, total phenolic compounds. In each row, different letters mean significant differences between samples (p < 0.05), and ^*^means significant differences between samples according to t-student test (p < 0.05).

Regarding the phenolic profile of leaf blades, 11 compounds were detected with four of them being identified as phenolic acids and six flavonoid glycoside derivatives and one other phenolic compound (Table 4). The most abundant compounds were luteolin-7-*O*-glucuronide (14.7 mg g^−1^ extract), 5-*O*-caffeoylquinic acid (13.38 mg g^−1^ extract), trans 3,5-*O*-dicaffeoylquinic acid (11.80 mg g^−1^ extract), and luteolin-7-*O*-glucoside (10.5 mg g^−1^ extract).

Concerning the phenolic profile of leaf midribs and petioles, the specific plant part was also characterized by the abundance of 5-*O*-caffeoylquinic acid (10.6 mg g^−1^ extract) and trans 3,5-*O*-dicaffeoylquinic acid (5.0 mg g^−1^ extract), although in different amounts comparing to cardoon heads (Table 4). Thirteen different phenolic compounds were identified, with nine being characterized as phenolic acids (20.33 mg g^−1^ extract) and four as flavonoids (4.07 mg g^−1^ extract).

Lastly, in what concerns cardoon seeds, two phenolic acids were identified being coincident to those detected in high concentrations in heads, leaf blades, and leaf midribs and petioles, with trans 3,5-*O*-dicaffeoylquinic acid (44.71 mg g^−1^ extract) as the most abundant compound, followed by 5-*O*-caffeoylquinic acid (4.25 mg g^−1^ extract) (Table 4).

### Antioxidant activity

Antioxidant activity of the various cardoon plant parts is presented in Table 5. Significant differences in antioxidant properties of the studied plant parts hydromethanolic extracts were observed (Table 5), with seeds and leaf blades showing the highest antioxidant potency for the various performed assays. In particular, seeds exhibited the highest scavenging activity for the DPPH and reducing power assays (EC_50_ values of 143 and 87 μg mL^−1^, respectively), while blades were the most efficient plant part for the β-carotene bleaching inhibition method (EC_50_ value of 114 μg mL^−1^). Lipid peroxidation inhibition assay (TBARS) did not show significant differences between leaf blades and seeds, which were the plant parts with the highest antioxidant activity (EC_50_ values of 112 and 125 μg mL^−1^, respectively). Although the differences in total phenolic compounds content between the various leaf parts could justify the results regarding their antioxidant activity (Tables 4, 5), this is not the case when comparing heads and leaf blades which contain similar amounts of phenolic.

**Table 5:** Antioxidant properties of hydromethanolic extracts of cardoon heads, leaf midribs and petioles, leaf blades, and seeds (EC_50_ values in μg mL^−1^; mean ± SD, *n* = 3).

**Sample**	**Radical scavenging activity**		**Reducing power**	**Lipid peroxidation inhibition**
	DPPH scavenging activity (EC_50_; mg/mL)^*^	**β-carotene/linoleate (EC_50_; mg/mL)**	**Ferricyanide/Prussian blue (EC_50_; mg/mL)**	**TBARS**
Heads	466 ± 5b	836 ± 32b	191 ± 1c	295 ± 8b
Leaf midribs and petioles	1238 ± 60a	9399 ± 282a	691 ± 11a	964 ± 18a
Leaf blades	218 ± 11c	114 ± 5d	273 ± 4b	112 ± 3c
Seeds	143 ± 1d	546 ± 30c	87 ± 1d	125 ± 3c

* *DPPH, 2,2-diphenyl-1-picrylhydrazyl; TBARS, thiobarbituric acid reactive substances*.

*The antioxidant activity was expressed as EC_50_ values, what means that higher values correspond to lower reducing power or antioxidant potential. EC_50_: Extract concentration corresponding to 50% of antioxidant activity or 0.5 of absorbance in reducing power assay. Trolox EC_50_ values: 41 μg/mL (reducing power), 42 μg/mL (DPPH scavenging activity), 18 μg/mL (β-carotene bleaching inhibition) and 23 μg/mL (TBARS inhibition). In each column, different letters mean significant differences between samples (p < 0.05)*.

### PCA analysis

Principal component analysis (PCA) is used to reduce multivariate data complexity as a method of identifying patterns and expressing data in ways that highlight similarities and differences, and further identify groups of samples or their geographical origin (Cheng et al., 2013, 2015). In the present study, PCA analysis was implemented in order to evaluate simultaneous changes in the nutritional profile and chemical composition patterns of cardoon plant parts. The first three axes of PCA explained 98.7% of total variation, indicating correct application of PCA to nutritional value and chemical composition cultivated cardoon plant parts and allowing differentiation between plant parts (Table S1 and Figure S3). Indeed, the first principal component identified discrete responses between the evaluated parameters, reaching a cumulative contribution ratio of 41.7%, while the second principal component showed a further separation of the ecotypes by 41.2%. The third principal component added a further variation of 15.8%. All the PCA components were statistically significant.

Micronutrients (Mn and Fe), Caffeic acid hexoside, p-Coumaric acid hexoside, 5-O-Feruloylquinic acid, Luteolin-7-O-rutinoside, Luteolin-7-O-glucuronide, Luteolin-7-O-glucoside, Pinoresinol-4-O-hexoside, total flavonoids, and other phenolic compounds were positively associated with the first principal component, whereas TBARS, β-carotene, DPPH, cis 3-O-Caffeoylquinic acid, 1,3-O-Dicaffeoylquinic acid, Eriodictyol-O-glucuronide, 4,5-O-Dicaffeoylquinic acid, Succinoyl-di-O-caffeoylquinic were negatively associated with the first principal component (Table S2).

Micronutrients (Na and Ca), Reducing power, trans 3,5-O-Dicaffeoylquinic and Luteolin-7-O-malonylglucoside were positively associated with the second principal component, whereas Mg and Zn, cis 3,5-O-Dicaffeoylquinic acid and Total phenolic acids were negatively associated with the second component (Table S2).

Finally, K, trans 3-O-Caffeoylquinic acid, 5-O-Caffeoylquinic acid, Luteolin-O-acetylglucuronide, cis 3,4-O-Dicaffeoylquinic acid, trans 3,4-O-Dicaffeoylquinic acid, Apigenin-O-glucuronide, and total phenolic compounds were all positively correlated with the third principal component (Table S2).

PCA clearly classified the studied cardoon plant parts which were differentiated according to their chemical composition and nutritional value (Figure S4).

## Discussion

### Nutritional value

Cardoon heads have a high nutritional value and can be considered a rich vegetable source of carbohydrates. Although wild cardoon is traditionally used as an edible vegetable in many local cuisines (heads and petioles), no reports regarding the nutritional value of cultivated cardoon genotypes are available. In our previous study, we evaluated the nutritional value of globe artichoke heads grown under similar conditions with the present study, including heads of two wild cardoon ecotypes (one with small and one with large spines in heads) (Petropoulos et al., in Press), and we reported similar results for nutritional value and sugars composition for the less spiny and more spiny ecotype, respectively. These differences between the wild cardoon ecotypes could be partly attributed to differences in the water content of heads that resulted in a concentration effect when results are expressed on a fresh weight basis, as well as to genotype effect (Petropoulos et al., in Press). Moreover, cultivation practices, harvesting stage as well the crop age may also affect chemical composition of cardoon plant parts (Lombardo et al., 2010; Pandino et al., 2011b).

### Chemical composition

Heads and seeds of cardoon are a good source of fatty acids, while seeds are also rich in oil which could be used for food and/or industrial uses. Similar seed oil yields with those of our study have been also recorded for various cardoon genotypes by Maccarone et al. (1999) who reported values between 24.9 and 25.6% (w/w) on a dry weight basis.

Regarding fatty acid composition, heads and seed oils contain 22 and 18 individual fatty acids, respectively, with palmitic and behenic acid being the most abundant fatty acids in seeds and linoleic and oleic acid in seed oil. Petropoulos et al. (in Press) who evaluated the chemical composition of heads of two wild cardoon ecotypes reported a significant effect of genotype on fatty acid composition, while the fatty acids profile of the evaluated ecotypes differed from the results of the present study with palmitic and linoleic acid being the main detected fatty acids. In addition, Maccarone et al. (1999) and Curt et al. (2002) reported a fatty acid composition for cardoon seed oils within the same range of the present study, with linoleic and oleic being the main fatty acids, followed by palmitic and stearic acid.

Fatty acids profile is of major importance and highlights the nutritional value of a food product. Although cardoon heads can be used for food purposes, the ratios of n-6/n-3 and PUFA/SFA do not indicate significant health benefits, in contrast with seed oils which show a high nutritional value and functional properties. Considering that Guil et al. (1996) and Simopoulos (2008) have highlighted the importance of both ratios for the nutritional value of a food product, only seed oils presented a health beneficial nutritional value with n-6/n-3 and PUFA/SFA ratios having values lower than 4.0 (4.94) and higher than 0.45 (0.57), respectively, which indicates the high potential of using this oil for human consumption. Simopoulos (2004) and Harnack et al. (2009) have demonstrated the pivotal role of long chain PUFA in human diet and n-6 and n-3 fatty acids in particular, while they also reported that ratios of 10:1 (n-6/n-3) are very common in Western diets and are highly associated with the formation of pro-inflammatory/aggregatory fatty acids such as eicosanoids. In contrast, cardoon heads exhibited values outside of thresholds for both ratios, a quality feature that detracts from the overall value of the edible part of the plant. Considering, the results of our previous study where two wild cardoon ecotypes were evaluated (Petropoulos et al., in Press), it seems that although fatty acids profile depends on the genotype, in neither case wild cardoon heads showed a high nutritional value in terms of their fatty acids composition.

All plant parts were rich sources of minerals and showed a high nutritional value with differences between plant parts regarding their content of the studied minerals. Slightly higher values regarding K content and lower content of Na and Ca in whole leaves of hydroponically grown cardoon plants have been reported by Borgognone et al. (2014), although they noted a significant effect of growth stage on mineral composition of leaves. In addition, Rouphael et al. (2012) detected similar amounts of K and Mg and lower content of Ca in cardoon plants grown in a floating culture system, while Colla et al. (2013) reported a significant variation in mineral composition of different cardoon genotypes grown under saline conditions. The differences of the present study with the already reported studies could be attributed to different growing systems (hydroponically grown vs. soil grown plants), growing conditions (greenhouse vs. field experiments), as well as to differences in plant age since the above-mentioned studies refer to young seedlings comparing to well established plants (5 years old) used in our study.

### Phenolic compounds

All cardoon plant parts are rich sources of phenolic compounds with significant differences in phenolic compounds profile. The detected compounds identification was previously described in samples of artichoke heads (Petropoulos et al., in Press), leaf midribs and petioles, and leaf blades (Petropoulos et al., 2017b).

The main compounds in cardoon heads were *trans* 3,5-*O*-dicaffeoylquinic acid and 5-*O*-caffeoylquinic, which have also been previously reported as the main compounds in heads of wild cardoon ecotypes (Petropoulos et al., in Press). The remaining phenolic compounds consisted mostly of caffeoylquinic acid and apigenin derivatives in a total of seven identified compounds. Ramos et al. (2014) reported similar amounts of total phenolic content in cardoon capitula (receptacle and bracts), while detecting 5-*O*-caffeoylquinic acid and 1,5-di-*O*-caffeoylquinic acid as the main phenolic compounds. In contrast, Pandino et al. (2010) detected only flavonoids and derivatives in cultivated cardoon heads, while in capitula of wild cardoon cultivar “Sylvestris Creta” phenolic acids were identified, thus, significantly lower amounts of 1,5-dicaffeoylquinic acid was detected. However, according to the study of Pandino et al. (2013) phenolic composition of globe artichoke receptacles exhibits a significant variation during the year with higher amounts of caffeoylquinic acid acids being detected in April; therefore, harvesting stage is essential for phenolic compounds composition. Moreover, genotypic and growing conditions differences, as well as extraction method may also play an important role in phenolic compounds profile and could justify the contrasting results of the reported studies (Pandino et al., 2012, 2013; Kollia et al., 2016). Pandino et al. (2013) have also stressed out the importance of weather conditions during harvest as well as the effect of harvesting time on chemical composition of globe artichoke.

The most abundant compounds in leaf blades were luteolin-7-*O*-glucuronide, 5-*O*-caffeoylquinic acid, trans 3,5-*O*-dicaffeoylquinic acid, and luteolin-7-*O*-glucoside, all of which have been previously reported by Petropoulos et al. (2017b). These results are in agreement with those of Ramos et al. (2014) and Pandino et al. (2011b) who studied phenolic compounds composition of intact cardoon leaves. Considering the bulky composition of cardoon leaves and the great diversity in leaf morphology between the various genotypes, the relative portions of blades, midribs and petioles could play an important role not only on total phenolic compounds content but also in phenolic compounds profile (Petropoulos et al., 2017b). Pinelli et al. (2007) have also confirmed the effect of environmental conditions on phenolic compounds content of wild and cultivated cardoon leaves.

Thirteen different phenolic compounds were identified in leaf midribs and petioles, with nine being characterized as phenolic acids and four as flavonoids. Similarly, Borgognone et al. (2014) detected higher amounts of chlorogenic acid and cynarin than flavonoids in cardoon leaves, while they also observed an increasing trend for phenolic acids with plant development. The compounds identified in the present study were also reported by Petropoulos et al. (2017b) in wild cardoon and globe artichoke ecotypes, while Pinelli et al. (2007) detected apart from chlorogenic and dicaffeoylquinic acid, luteolin-7-*O*-glucoside, luteolin-7-*O*-malonylglucoside and luteolin-7-*O*-glucuronide in amounts that depended on genotype and post-harvest processing (blanching). Lower amounts of flavonoids (mainly luteolin) than chlorogenic acid and cynarin in cardoon leaves have been also reported by Rouphael et al. (2012), who also suggested a negative correlation of phenolic compounds content and nutrient solution concentration. In contrast, Ramos et al. (2014) suggested that cardoon leaves had the lowest total phenolic compounds content among the various plant parts (stalks and capitula), with flavonoids being the predominant class of phenolic compounds (98% of TPC). The same trend was also observed by Pandino et al. (2011b) who suggested flavonoids as the main class of phenolic compounds, with a significant genotypic variation being reported, whereas Juániz et al. (2016) detected only chlorogenic acid and its derivatives, and traces of flavonoids in cardoon stalks. These discrepancies in the literature could be attributed not only to genotypic effect but also to harvest date since according to Wang et al. (2003) significant differences in phenolic compounds composition were observed in leaves harvested at two different dates. Other factors that may be involved in polyphenols composition are environmental conditions and cultivation management practices (Pinelli et al., 2007; Pandino et al., 2011a,b).

Seeds contain only two individual phenolic compounds which were identified as 3,5-*O*-dicaffeoylquinic acid and 5-*O*-caffeoylquinic acid. These compounds were, as referred above, previously described by our research group in globe artichoke and wild cardoon samples (Petropoulos et al., 2017b, in Press). Moreover, Khaldi et al. (2013) studied several cultivars of wild and cultivated Tunisian cardoon seeds and reported the total polyphenol content of methanolic extracts (23.25 and 15.04 mg GAE g^−1^ dw, respectively), as also the flavonoid (8.93 mg CE g^−1^ dw) and tannin (4.62 mg CE g^−1^ dw) contents of the wild cardoon seeds extracts. In another study, Falleh et al. (2008) reported lower amounts of total polyphenol (14.33 mg GAE g^−1^ dw) and tannins (2.00 mg CE g^−1^ dw) in cardoon seeds, but a higher concentration of flavonoids (9.78 mg CE g^−1^ dw).

### Antioxidant activity

Antioxidant activity differed between the various plant parts, with seeds exhibiting the highest potency. Antioxidant potential of cardoon plant parts could be partly attributed to specific polyphenols, as well as to other bioactive molecules, such as tannins and saponins (Durazzo et al., 2013; de Falco et al., 2015; Sihem et al., 2015), depending on the extraction method (Kukić et al., 2008; Brás et al., 2015; Kollia et al., 2016). Differences in antioxidant properties of wild cardoon morphological organs have been also reported by Petropoulos et al. (2017b) and Petropoulos et al. (in Press), although they suggested that heads were the most potent organs comparing to leaf blades and midribs and petioles. Similarly, in the study of Falleh et al. (2008) seeds showed higher antioxidant potential than leaves and flowers. In addition, Durazzo et al. (2013) evaluated antioxidant properties of various cultivated and wild cardoon genotypes and reported significant differences between cultivated cardoon for the ferric reducing antioxidant power (FRAP) assay, while Trolox equivalent antioxidant capacity (TEAC) values differed significantly for both cultivated and wild genotypes. Finally growing conditions and cultivation practices may also effect antioxidant activities of cardoon plants parts, since they play an important role in phenolic compounds composition which are considered as potent antioxidant compounds (Moglia et al., 2008; Lombardo et al., 2009; Colla et al., 2013).

## Conclusion

Cardoon is an important multipurpose field crop of the Mediterranean basin which is primarily proposed for energy and solid biofuel production. However, numerous health effects and medicinal properties have been attributed to the various plant parts, since they contain significant amounts of bioactive molecules. The results of the present study showed that apart from industrial uses, cardoon plant parts may also find alternative uses in the food and pharmaceutical industry, either as vegetable products (immature heads) and ingredients in functional foods and herbal medicines or as sources of bioactive molecules. Moreover, seed oils exhibited a very nutritious profile which could be further valorized for the production of alternative vegetable oils and herbal formulations for human consumption.

## Author contributions

SP and IF: Designed the experiments; CP and LB: Performed the analytical assays and analyzed the data; SP, NT, and LB: Wrote the manuscript; SP, NT, and IF: Revised the manuscript.

### Conflict of interest statement

The authors declare that the research was conducted in the absence of any commercial or financial relationships that could be construed as a potential conflict of interest.
