# Supplementary material for: Nutritional Value and Bioactive Compounds Characterization of Plant Parts From Cynara cardunculus L. (Asteraceae) Cultivated in Central Greece
Source: Front Plant Sci. 2018 Apr 10;9:459. doi: 10.3389/fpls.2018.00459 (PMC5902674; doi:10.3389/fpls.2018.00459)
Supplement: Supplementary file 1 [file DataSheet1.docx]

**Figure S3.** Three dimensional principal component analysis (PCA) illustrating the variation of chemical composition and nutritional value of cultivated cardoon plant parts.

Leaf blades

Petioles and midribs

Heads

Seeds

**Figure S4.** 3D scatterplot of the main sources of variability between the cardoon plant parts.

**Abbreviations of the variables:**

cis 3-O-Caffeoylquinic acid: 3-O-CA

trans 3-O-Caffeoylquinic acid : t3-O-CA

Caffeic acid hexoside: CAH

p-Coumaric acid hexoside: p-CAH

5-O-Caffeoylquinic acid: 5-O-CA

1,3*-O-*Dicaffeoylquinic acid: CYN

Luteolin-O-acetylglucuronide: L-O-AG

5-O-Feruloylquinic acid: 5-O-FA

Eriodictyol-O-glucuronide: E-O-G

Luteolin-7-O-rutinoside: L-7-O-R

cis 3,4-O-Dicaffeoylquinic acid: c3,4-O-DCQA

Luteolin-7-O-glucuronide: L-7-O-Gr

trans 3,4-O-Dicaffeoylquinic acid: t3,4-O-DCQA

Luteolin-7-O-glucoside: L-7-O-Gs

Pinoresinol-4-O-hexoside: P-4-O-H

cis 3,5-O-Dicaffeoylquinic acid: c3,5-O-DCQA

trans 3,5-O-Dicaffeoylquinic: t3,5-O-DCQA

4,5-O-Dicaffeoylquinic acid: 4,5-O-DCQA

Succinoyl-di-O-caffeoylquinic: SD-O-CQA

Apigenin-O-glucuronide: A-O-G

Luteolin-7-O-malonylglucoside: L-7-O-MG

Total phenolic acids: TPA

Total flavonoids: TF

Other phenolic compounds: OPC

Total phenolic compounds: TPC

**Table S1.** Principal component analysis

| *Component* |  | *Percent of* | *Cumulative* |
| --- | --- | --- | --- |
| *Number* | *Eigenvalue* | *Variance* | *Percentage* |
| 1 | 15,0155 | 41,710 | 41,710 |
| 2 | 14,8399 | 41,222 | 82,932 |
| 3 | 5,67894 | 15,775 | 98,706 |
| 4 | 0,285073 | 0,792 | 99,498 |
| 5 | 0,0808294 | 0,225 | 99,723 |
| 6 | 0,0425359 | 0,118 | 99,841 |
| 7 | 0,0347305 | 0,096 | 99,937 |
| 8 | 0,0124127 | 0,034 | 99,972 |
| 9 | 0,0064984 | 0,018 | 99,990 |
| 10 | 0,00308549 | 0,009 | 99,999 |
| 11 | 0,000511554 | 0,001 | 100,000 |
| 12 | 1,44944E-15 | 0,000 | 100,000 |
| 13 | 1,05017E-15 | 0,000 | 100,000 |
| 14 | 9,94545E-16 | 0,000 | 100,000 |
| 15 | 7,20838E-16 | 0,000 | 100,000 |
| 16 | 7,18235E-16 | 0,000 | 100,000 |
| 17 | 4,91117E-16 | 0,000 | 100,000 |
| 18 | 4,58388E-16 | 0,000 | 100,000 |
| 19 | 4,35473E-16 | 0,000 | 100,000 |
| 20 | 2,05109E-16 | 0,000 | 100,000 |
| 21 | 1,04257E-16 | 0,000 | 100,000 |
| 22 | 5,00696E-17 | 0,000 | 100,000 |
| 23 | 1,42145E-17 | 0,000 | 100,000 |
| 24 | 0,0 | 0,000 | 100,000 |
| 25 | 0,0 | 0,000 | 100,000 |
| 26 | 0,0 | 0,000 | 100,000 |
| 27 | 0,0 | 0,000 | 100,000 |
| 28 | 0,0 | 0,000 | 100,000 |
| 29 | 0,0 | 0,000 | 100,000 |
| 30 | 0,0 | 0,000 | 100,000 |
| 31 | 0,0 | 0,000 | 100,000 |
| 32 | 0,0 | 0,000 | 100,000 |
| 33 | 0,0 | 0,000 | 100,000 |
| 34 | 0,0 | 0,000 | 100,000 |
| 35 | 0,0 | 0,000 | 100,000 |
| 36 | 0,0 | 0,000 | 100,000 |

**Table S2.** Classification of the estimated parameters in three principal components**.**

|  | *Component* | *Component* | *Component* |
| --- | --- | --- | --- |
|  | 1 | 2 | 3 |
| K | -0,0505258 | 0,0870459 | 0,3819 |
| Na | -0,0905345 | 0,217821 | 0,129808 |
| Ca | -0,0663663 | 0,237912 | 0,104453 |
| Mg | -0,0853531 | -0,195404 | -0,167305 |
| Mn | 0,235352 | -0,0887689 | -0,0613098 |
| Fe | 0,194495 | 0,147853 | 0,0574261 |
| Zn | 0,084344 | -0,227947 | 0,133976 |
| TBARS | -0,218008 | 0,117009 | 0,119668 |
| RP | -0,158794 | 0,185454 | 0,139279 |
| β-carotene | -0,213785 | 0,139181 | 0,0651321 |
| DPPH | -0,20924 | 0,115951 | 0,158416 |
| 3-O-CA | -0,204854 | 0,153508 | 0,0590627 |
| t3-O-CA | 0,0770206 | -0,123866 | 0,346701 |
| CAH | 0,21576 | 0,13816 | 0,0488253 |
| p-CAH | 0,216158 | 0,138336 | 0,0485414 |
| 5-O-CA | 0,0287082 | -0,116029 | 0,372273 |
| CYN | -0,204801 | 0,153535 | 0,0591645 |
| L-O-AG | -0,00424367 | -0,193311 | 0,279921 |
| 5-O-FA | 0,216019 | 0,138306 | 0,0487914 |
| E-O-G | -0,204855 | 0,15351 | 0,059067 |
| L-7-O-R | 0,21622 | 0,138395 | 0,0486458 |
| c3,4-O-DCQA | -0,0839733 | -0,145298 | 0,319335 |
| L-7-O-Gr | 0,202826 | 0,156691 | 0,0555975 |
| t3,4-O-DCQA | -0,00423993 | -0,193301 | 0,279924 |
| L-7-O-Gs | 0,216122 | 0,138308 | 0,048508 |
| P-4-O-H | 0,216079 | 0,138275 | 0,0484725 |
| c3,5-O-DCQA | -0,0123099 | -0,247147 | -0,125977 |
| t3,5-O-DCQA | 0,13674 | 0,21474 | 0,0778152 |
| 4,5-O-DCQA | -0,204801 | 0,153412 | 0,0589494 |
| SD-O-CQA | -0,204812 | 0,153427 | 0,0589645 |
| A-O-G | -0,00421717 | -0,193164 | 0,279837 |
| L-7-O-MG | 0,179632 | 0,181771 | 0,0652212 |
| TPA | 0,028614 | -0,251347 | 0,0932688 |
| TF | 0,207807 | 0,135461 | 0,117793 |
| OPC | 0,216079 | 0,138275 | 0,0484725 |
| TPC | 0,168327 | -0,166317 | 0,169524 |
